# Time Domains of Hypoxia Adaptation—Elephant Seals Stand Out Among Divers

**DOI:** 10.3389/fphys.2019.00677

**Published:** 2019-06-04

**Authors:** Michael S. Tift, Paul J. Ponganis

**Affiliations:** ^1^Department of Biology and Marine Biology, University of North Carolina Wilmington, Wilmington, NC, United States; ^2^Center for Marine Biotechnology and Biomedicine, Scripps Institution of Oceanography, University of California, San Diego, La Jolla, CA, United States

**Keywords:** blood oxygen transport, carbon monoxide, dive, hemoglobin, hypoxia tolerance, lung, myoglobin, ischemia reperfusion injury

## Introduction

In this Opinion, adaptations to hypoxia are examined during the short time domains of breath holds from three accomplished diving animals: northern elephant seals (*Mirounga angustirostris*), California sea lions (*Zalophus californianus*), and emperor penguins (*Aptenodytes forsteri*). Review of dive behavior, oxygen (O_2_) storage, and arterial blood O_2_ profiles during dives reveals that the elephant seal undergoes the most frequent and extreme hypoxemia. Exceptional breath hold durations, routine hypoxemia, established research protocols, and accessibility to the animals make the elephant seal stand out for physiological investigation and evaluation of biochemical/molecular adaptations in hypoxemic tolerance, protection against re-perfusion injury, and O_2_ transport during dives.

The northern elephant seal and southern elephant seal (*M. leonina*) are the premier pinniped divers (Le Boeuf et al., 1988; Hindell et al., 1991, 1992; Hassrick et al., 2010; Robinson et al., 2012). During several month-long trips to sea, these animals spend 80–90% of their time underwater, perform routine dives of 20–30 min duration to average depths >400 m, have short inter-dive surface intervals that average two min, and typically gain about one kg d^−1^ in body mass.

In contrast to elephant seals, California sea lions only spend about 30% of their time at sea diving (Feldkamp et al., 1989). Most dives are < 100 m in depth and between 1 and 4 min in duration (McDonald and Ponganis, 2013; Tift et al., 2017). However, dependent on geographic location, climate variability, and prey distribution, these sea lions can regularly perform 10-min dives to 400–500 m, with the current longest reported dive of 16 min (Melin et al., 2008; McHuron et al., 2016, 2018).

Emperor penguins are the premier avian divers; they exploit the entire water column to depths >500 m. Shallower dives are up to 5–6 min in duration while deep dives are about 10 min (Kooyman and Kooyman, 1995; Kirkwood and Robertson, 1997; Sato et al., 2011). The longest dive documented by a continuous dive profile in an emperor penguin is 27.6 min. During foraging trips to sea, emperor penguins spent about 31% of their time resting on the sea ice (Watanabe et al., 2012).

## O_2_ Stores: Magnitude, Distribution and Utilization

The magnitude and distribution of respiratory, blood, and muscle O_2_ stores are dependent on diving lung volume, blood volume, hemoglobin (Hb) concentration, muscle mass, myoglobin (Mb) concentration, and the quantity of extractable O_2_ from each store (i.e., reduction in Hb saturation during a dive). As recently reviewed (Ponganis, 2015), total body O_2_ stores have been estimated at 94, 55, and 68 ml O_2_ kg^−1^ body mass for elephant seals, California sea lions, and emperor penguins, respectively. The distribution of these stores varies considerably with 68, 39, and 31% in the blood, 28, 48, and 36% in muscle, and 4, 13, and 33% in the respiratory system of elephant seals, California sea lions, and emperor penguins, respectively.

The cardiovascular dive response (the decrease in heart rate (bradycardia) and increase in peripheral vascular resistance associated with a breath hold) and pulmonary gas exchange play a critical role in blood O_2_ utilization and arterial partial pressure of O_2_ (P_O2_) profiles during dives. Heart rate is a primary determinant of pulmonary blood flow and, consequently, extraction of O_2_ from the lung. Vasoconstriction and redistribution of peripheral blood flow which accompany the bradycardia result in a decreased blood O_2_ extraction by tissue, thus slowing the depletion rate of the blood O_2_ store (Irving et al., 1941; Valtin, 1973; Lutz et al., 1975; Kvietys and Granger, 1982). Elephant seals, California sea lions, and emperor penguins all display variability in the degree of diving bradycardia which can be dependent on the depth and nature of a given dive (Andrews et al., 1997; Meir et al., 2008; McDonald and Ponganis, 2014; Wright et al., 2014).

Cessation of gas exchange at depth due to alveolar collapse (100% pulmonary shunt) also affects arterial oxygenation patterns during dives in marine mammals (Kooyman et al., 1970, 1973a; Kooyman and Sinnett, 1979, 1982; Falke et al., 1985; Fahlman et al., 2009, 2017; McDonald and Ponganis, 2012). In penguins, it is unclear if gas exchange ceases at depth (Kooyman et al., 1973b).

## Arterial P_O2_ and Hemoglobin Saturation Profiles During Dives

**Figure 1:**
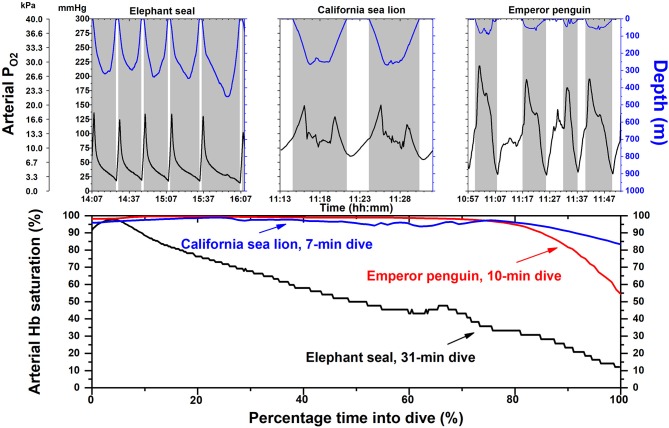
Arterial partial pressure of oxygen (P_O2_) and hemoglobin (Hb) saturation profiles in elephant seals, California sea lions and emperor penguins. Depth and P_O2_ profiles are depicted for each species in the upper panel. The shape of the arterial P_O2_ profiles of individual dives are dependent on the magnitude and distribution of O_2_ stores in each animal, depth of dive, and on multiple factors within a dive, including heart rate, lung O_2_ extraction, peripheral O_2_ delivery (i.e., the intensity of the dive response), lung or air sac volume and compression with depth, and the depth at which pulmonary gas exchange ceases in mammals (alveolar collapse, i.e., 100% pulmonary shunt). Shaded areas indicate dives; time scale is different for each species. In the lower panel, Hb saturation profiles for a single dive in each species were constructed with their respective O_2_-Hb dissociation curves and an arterial P_O2_ profile. In elephant seals, arterial Hb saturation is below 80% for most of the dive. In contrast arterial Hb saturations in sea lions and emperor penguins remain above 90% for most of the dive. Time scale is percentage of time of total dive duration. Adapted from prior publications (Meir and Ponganis, 2009; Meir et al., 2009; McDonald and Ponganis, 2012, 2013).

Arterial P_O2_ profiles and calculated Hb saturation profiles have been obtained during dives of these three species (Figure 1) with use of intravascular P_O2_ electrodes, backpack bio-loggers, and O_2_-Hb dissociation curves on free-diving animals (Meir and Ponganis, 2009; Meir et al., 2009; McDonald and Ponganis, 2012, 2013; Tift et al., 2017, 2018). Among these three elite divers, it is the elephant seal that experiences routine and extreme arterial hypoxemia with arterial Hb saturations below 80% for ~80% of dive durations (Figure 1). Although hypoxemia likely occurs in the other two species, it is notable that their arterial Hb saturations can remain above 90% for almost 90% of the dive duration (Figure 1). In sea lions and penguins, the maintenance of high arterial Hb saturations throughout much of the dive are attributable, at least in part, to (a) diving on inspiration not expiration (Sato et al., 2002; Fahlman et al., 2008; McDonald and Ponganis, 2012; Tift et al., 2017), (b) larger respiratory fraction of total O_2_ stores, and (c) maintenance of gas exchange at deeper depths.

The elephant seal also experiences significant arterial Hb desaturation during its spontaneous, frequent sleep apneas on land (Stockard et al., 2007). All these studies reveal that elephant seals encounter hypoxemia far more often in their life cycle than either California sea lions or emperor penguins. Based upon the Krogh Principle (Krebs, 1975), elephant seals are ideal models for investigation of the physiological and biochemical mechanisms of hypoxemic tolerance in divers.

## Cerebral Hypoxemic Protection

In general, there are three factors in seals that contribute to enhanced brain O_2_ delivery during breath holds: (a) high Hb concentrations, (b) hypercarbia (leading to decreased Hb-O_2_ affinity and an increase in cerebral blood flow, and (c) increased brain capillary density (Kerem and Elsner, 1973). In addition, selective brain cooling and potential cerebro-protection can occur via arterio-venous shunting in the foreflippers with brain temperature declines of 3–4°C during 15-min forced submersions (Blix et al., 2010). Some notable biochemical and molecular adaptations in the seal brain include: (a) a 3-fold elevation in brain glycogen, (b) normal lactate dehydrogenase (LDH) activity with >70% LDH 1 and 2 isoenzymes (lactate oxidation), primarily located in glial cells, (c) increased gene expression of S100B (a stress protein with calcium binding activity), clustrin (an extracellular chaperone molecule), and most glycolytic enzymes, but decreased expression of pyruvate dehydrogenase, and (d) normal neuroglobin and cytochrome oxidase gene expression, but, in contrast to terrestrial mammals, located in glial cells (Mitz et al., 2009; Schneuer et al., 2012; Czech-Damal et al., 2014; Fabrizius et al., 2016; Hoff et al., 2016, 2017). The ability to study elephant seals during their voluntary sleep apnea events, which can include routine ten-min breath holds even inside an NMR scanner (Ponganis et al., 2008), make them ideal model organisms to investigate metabolic rate, glucose consumption, and blood flow in brain and muscle with advanced scanning techniques, such as functional magnetic resonance imaging, positron emission tomography, and near-infrared diffuse correlation spectroscopy (Ridgway et al., 2006; Smith et al., 2013; Shang et al., 2017).

## Avoidance of Re-perfusion Injury

Re-perfusion injury occurs when O_2_-rich blood returns to previously ischemic and hypoxic tissues, and is often associated with reactive oxygen species (ROS) generation, intracellular calcium accumulation, and inflammation (Powers and Jackson, 2008). In the seal heart, although a 10-fold elevation in glycogen content may provide a large glycolytic energy store and prevent intracellular calcium accumulation during ischemia/hypoxemia, an impressive 25-fold elevation in glutathione content should also enhance the potential for scavenging of ROS during re-perfusion (Henden et al., 2004; Vázquez-Medina et al., 2007). Significant elevations in glutathione content were also found in seal kidney, lung, and muscle. In all tissues, enzymes associated with the recycling of glutathione were elevated. In addition to enhanced scavenging of ROS, it has also been found that the whole blood inflammatory response of seals on exposure to a potent endotoxin (lipopolysaccharide—LPS) is significantly blunted (Bagchi et al., 2018). Interleukin-6 cytokine production in blood was 50–500 times lower in elephant seals and Weddell seals (*Leptonychotes weddellii*) than in humans. Lastly, endogenous carbon monoxide (CO) levels are high in these two species with carboxyhemoglobin levels as high as 10% in elephant seals (Pugh, 1959; Tift et al., 2014). Such high CO levels raise the possibility that CO may play a role in the prevention of inflammatory responses during re-perfusion. Exposure to moderate levels of exogenous CO has shown to exhibit potent anti-inflammatory effects (Motterlini and Otterbein, 2010). Again, elephant seals represent ideal models for further investigation in re-perfusion injury avoidance and the physiological role of endogenous CO, with established protocols in place to examine tissue stress responses and collect serial blood samples during voluntary breath-holds (Stockard et al., 2007; Vazquez-Medina et al., 2011a,b; Tift et al., 2013, 2014).

## O_2_ Transport: Hemoblobin O_2_ Affinity

Although the presence of carboxyhemoglobin should decrease the start-of-dive blood O_2_ store of the elephant seal, the increased Hb-O_2_ affinity induced by the presence of CO may be beneficial for O_2_ delivery during dives. Increased Hb-O_2_ affinity is advantageous during severe hypoxia because it promotes O_2_ uptake from the lung and increases the O_2_ content of blood at a given arterial P_O2_. The Hb-O_2_ affinity is increased in penguins and in a variety of mammals and birds adapted to live in hypoxic environments or at high altitude (Milsom et al., 1973; Black and Tenney, 1980; Weber, 2007; Meir and Ponganis, 2009; Storz et al., 2010; Storz, 2016; Weber et al., 2017). In many pinnipeds, including elephant seals, the Hb-O_2_ affinity is not known to be high; their P_50_ values (P_O2_ at 50% Hb saturation: low values = high Hb-O_2_ affinity) were 25–30 mm Hg (3.3–4.0 kPa) which resemble values seen in hypoxia intolerant species (Lenfant et al., 1969, 1970; Qvist et al., 1981; Meir et al., 2009; McDonald and Ponganis, 2013). However, in several species of cetaceans, high Hb-O_2_ affinities have been reported, with P_50_ values ranging from 19 to 25 mm Hg (2.5–3.3 kPa) (Horvath et al., 1968; Dhindsa et al., 1974; Vedvick and Itano, 1976). In the manatee, the P_50_ was near 16 mm Hg (2.1 kPa) (White et al., 1976; Farmer et al., 1979). The lack of a relative increase in Hb-O_2_ affinity of pinnipeds in contrast to that in other divers (penguins, cetaceans, manatees) warrants further investigation of carboxyhemoglobin levels, blood O_2_ contents, O_2_-Hb binding characteristics in the presence vs. absence of CO, and blood-to-tissue O_2_ transfer. The elephant seal is ideal with its high CO values, long dives and sleep apneas, its established research/blood sampling protocols (translocation studies at sea and sleep apnea studies on land), and its accessibility for research (Stockard et al., 2007; Ponganis et al., 2008; Meir et al., 2009; Tift et al., 2013).

## Conclusions

Elephant seals, sea lions, and emperor penguins are all highly adapted to perform remarkable dives. However, it is the elephant seal that undergoes the most frequent and extreme arterial hypoxemia due to its continuous dive behavior and sleep apneas on land. These factors, in addition to (a) extensive knowledge of biochemical and molecular adaptations to hypoxia in seals, (b) established sleep apnea and dive research protocols, and (c) its accessibility on the California coast make the elephant seal stand out for investigation of time domains of hypoxia adaptation in diving animals.

## Data Availability

The datasets for this manuscript are not publicly available because they have not been uploaded to a public database. Requests to access the datasets should be directed to pponganis@ucsd.

## Author Contributions

MT and PP wrote sections of the manuscript, contributed to manuscript revision, read, and approved the submitted version.

### Conflict of Interest Statement

The authors declare that the research was conducted in the absence of any commercial or financial relationships that could be construed as a potential conflict of interest.
